# Serum Adiponectin Level and Different Kinds of Cancer: A Review of Recent Evidence

**DOI:** 10.5402/2012/982769

**Published:** 2012-11-18

**Authors:** Vajihe Izadi, Elaheh Farabad, Leila Azadbakht

**Affiliations:** ^1^Food Security Research Center, Isfahan University of Medical Science, P.O. Box 81745, Isfahan, Iran; ^2^Departmant of Community Nutrition, School of Nutrition and Food Science, Isfahan University of Medical Science, P.O. Box 81745, Isfahan, Iran

## Abstract

*Background*. Adiponectin, an adipokine secreted from adipose tissue, has antiobesity, anti-insulin resistance, and anticancer roles. The present study aimed to review the epidemiologic evidence about the association between adiponectin and cancers. *Method*. We searched in PubMed from 2002 to October 2011 by using the following key words: cancer, malignancy, cell proliferation, and adiponectin. Finally, 45 articles were recruited to review in the present paper. *Findings*. Several findings suggested inverse association between concentration of hormone and breast cancer risk. Low levels of adiponectin increase the risk of endometrial cancer in women. Adiponectin levels were significantly associated with prostate cancer in men. It seems that there is an inverse relationship between levels of adiponectin or its gene and colorectal cancer. Significant association between hormone and pancreatic cancer was found. *Conclusion*. Several findings suggested the negative correlation between adiponectin and risk of cancers. This relationship was more elucidated by the correlation between the hormone with obesity and insulin resistance. Suppression of growth and proliferation of cancer cells by adiponectin were explained via several mechanisms.

## 1. Introduction

Obesity, influenced by environmental and genetic factors, is one of the important factors in the etiology of metabolic syndrome, cardiovascular disease (CVD), and cancer [1–3]. Obesity is related to increased level of inflammatory markers such as CRP (C-reactive protein) that are associated with metabolic syndrome [4]. According to evidence of 2005, 937 and 396 million people around the world were obese and overweight, respectively [5]. Obesity is significantly correlated with an increase in dietary energy density and also it can elevate the risk of CVD and metabolic syndrome [6, 7]. Adherence to the healthy dietary pattern [8] and high intake of fruits and vegetables [9] are inversely related to metabolic syndrome. Moreover, the consumption of plant proteins, such as beans, drives a significant reduction in the inflammatory markers such as CRP, while meat consumption can increase inflammatory markers in bloodstream [10, 11]. Dietary intakes have major role in controlling the inflammation and also weight management [12]. Obesity increases the risk of cancer. A study showed that the risk of cancer in obese women is 50% more than women with normal weight [13]. According to published studies, obesity, inflammation, insulin resistance, metabolic syndrome, cardiovascular disease, and cancer are significantly associated with concentrations of adiponectin [14–16].

Adiponectin is a peptide with 244 amino-acids, secreted from adipose tissue [17]. Plasma adiponectin levels in normal healthy individuals are 10 *μ*g/mL where it accounts 0.01% of all plasma proteins [18]. This hormone presents in plasma as two important epimers: low molecular weight (LMW) or trimeric complex and high molecular weight (HMW) or oligomeric complex [15, 18]. Adiponectin inhibits vascular smooth muscle proliferation to prevent atherosclerosis. It also has an impact on pathogenesis of diabetes through the regulation of glucose and free fatty acid metabolisms and insulin sensitivity in the epithelial cells [19, 20]. Two isoforms of adiponectin receptors were identified as Adipo R_1_ and Adipo R_2_. Adipo R_1_ receptors are predominantly expressed in muscle and Adipo R_2_ receptors are mainly expressed in the liver [15]. Although the hormone is secreted from adipose tissue, as the weight increases the plasma level of adiponectin decreases. Moreover weight loss diets can elevate the level of adiponectin in plasma [21]. Higher adiponectin concentrations in women are more likely due to estrogen or androgen in their bloodstream and are independent of fat mass [22]. While testosterone in men causes a reduction in concentrations of serum adiponectin and prevents production of HMW in adipose tissue [23]. 

Several studies suggest that individuals with higher levels of adiponectin show generally higher concentrations of high density lipoprotein (HDL) and lower levels of low density lipoprotein (LDL), triglycerides (TG), and total cholesterol [24]. This hormone also plays an important role in the secretion of estrogen and insulin-like growth factor (IGF), which are important cancer risk factors [25]. Studies indicate that cancer cells express adiponectin receptors thus attaching adiponectin to its receptors may limit the proliferation of cancer cells [18, 26]. Since obesity and insulin resistance are risk factors of cancer, adiponectin may act as an anticancer agent specially in breast cancer due to its significant effect on obesity and insulin resistance [15]. The relationship between adiponectin concentrations and prevention of cancer cells proliferation and its anticancer role [1, 27], and also the concentration of this hormone in patients with different types of cancers such as endometrial, breast, prostate, and colorectal cancer, has been investigated in several recent studies [26–29] which indicates a close relationship between adiponectin and types of cancers. Most studies [1, 27] but not all of them [29] suggest that the relationship between adiponectin and cancer is correlated with hormones, including estrogen, IGF_1_, obesity, and insulin resistance. Further investigations are needed to clarify the mechanisms of this relationship. This present study aims to review available evidence related to the serum adiponectin and breast, prostate, endometrial, and gastrointestinal tract cancers. 

## 2. Methods

In order to examine the relationship between serum adiponectin levels and breast cancer, endometrial cancer, prostate cancer, and colorectal cancer, 153 articles between 2002 and October 2011 were accessed by using PubMed search engine and using keywords such as: cancer, malignancy, cell proliferation, and adiponectin. 

45 articles with case control, cross-sectional, and prospective cohort design were reviewed. Other articles were excluded owing to lack of direct relation with the issue, duplication, and lack of access to full text articles. Studies that investigated among association between levels of adiponectin and cancer are shown in Table 1.

### 2.1. Relationship between Breast, Endometrial Cancers, and Concentrations of Adiponectin

Findings from several studies indicate an inverse relationship between serum adiponectin levels and breast cancer [18, 19, 26]. In a case control study on comprising 102 women with primary breast cancer patient and 100 healthy women, in the age of 51–55 years, those in the lowest tertile of serum adiponectin levels (≤9.6 *μ*g/mL) had a significantly higher risk of having breast cancer compared with women in the highest tertile (≥10.6 *μ*g/mL) [20]. This relationship was observed in women both in pre- and postmenopause (*P* < 0.005). Another study showed, that risk of breast cancer in cases who were in the highest quartile of serum adiponectin levels were 77% less than those who were in the lowest quartile of hormone (*P* = 0.02). Patients with cancer had 6.21% and 3.12% lower levels of adiponectin and HMW, respectively [26]. There was no significant relationship between hormone levels and risk of breast cancer in pre- and postmenopausal women (*P*_trend_ = 0.09 and *P*_trend_ = 0.08, resp.) [31]. In another case-control study on women with cancer and healthy women, aged 29–65 years, adiponectin levels in the cases were significantly (*P* < 0.005) lower than the controls. Also, obesity and physical inactivity can increase risk of breast cancer by 3 and 2–5 times, respectively. Low levels of adiponectin, obesity, and physical inactivity were reported as important risk factors for breast cancer in Malaysian women [19]. The presence of adiponectin in breast epithelial cells possibly has protective effects against cancer [32]. Study on 41 women with cancer and 45 control women suggested that serum adiponectin levels in the cases were nonsignificantly lower than control group (*P* = 0.37). Mean BMI in cases and controls was 23 [2]. 15.6% reduction in proliferation of MCF-7 breast cancer cells was reported by 10 *μ*g/mL adiponectin levels, during the 96 hours, in comparison with controls [17]. To conclude, the findings of different studies suggest that risk of breast cancer in low adiponectin concentrations increases independent of BMI, leptin, IGF1, and menopausal status [19, 20].

According to a survey in 2004, higher BMI and lower serum adiponectin levels increased risk of endometrial cancer, 6.5 times, more than women with normal BMI and high adiponectin concentrations [33]. In this study, adiponectin was considered as a mediator to explain the relationship between insulin resistance, obesity, and endometrial cancer. In a prospective nested case-control study which was conducted on over 284 women with endometrial cancer and 548 healthy women, those who had the highest adiponectin levels were significantly at lower risk of endometrial cancer compared with the women with the lowest levels of hormone (*P*_trend_ = 0.006). This relationship was stronger in obese women [29]. Also women with the lowest concentrations of adiponectin were at risk of endometrial cancer 11 fold more than women who have the highest level of the hormone [34].

### 2.2. Relationship between Serum Adiponectin and Prostate Cancer

The results of most epidemiological studies suggest an inverse relationship between hormone levels and risk of prostate cancer [28, 42]. In a prospective study on 654 men with prostate cancer and 644 healthy men, the cases in the highest quintile of serum adiponectin had lower risk of fatal prostate cancer, compared with the lowest quintile of adiponectin concentration [43]. This relationship was not substantial after adjusting the effect of BMI and C-peptide. Based on this study, adiponectin is considered as a possible mediator between obesity and prostate cancer [43]. In another study on 25 patients with benign prostatic hyperplasia and 43 prostate cancer patients, there was not any significant difference between hormone levels in both groups [44]. HMW may be considered as a growth inhibitor for proliferation and prostate cancer cells [45]. Also in a study, serum adiponectin was called as a potential antiprostate cancer [46]. In another case-control study, adiponectin concentrations in patients with prostate cancer were less than controls [39]. Different types of adiponectin gene in African American men with prostate cancer, who participated in a community-based case-control study, did not show significant association with prostate cancer [37].

### 2.3. Relationship between Adiponectin Levels and Gastrointestinal Tract Cancer

It seems that there is an inverse relationship between serum adiponectin concentrations, colon, and colorectal cancer [30, 47, 48]. In a cohort study, low levels of adiponectin were considered as a more important risk factor than BMI and TG in early stage of colorectal cancer [30]. This hormone was not a significant risk factor for advanced colorectal cancer. The risk of colorectal cancer in men who had high plasma adiponectin concentrations was 60% lower than those with lower levels of adiponectin after adjusting for some potential confounding (BMI, waist circumference, and physical activity) [47]. In a nested prospective case-control study on 381 men with colorectal cancer and 381 healthy men, no significant association between hormone levels and colorectal cancer was found [48]. On the other hand, in another study on men and women aged 48–80 with colon cancer, with and without cachexia, a higher level of adiponectin was observed in those with 5% or more BMI reduction in 6 months [49]. In this study no correlation was observed between weight loss and adiponectin levels. Results from several case-control colorectal cancer studies represent important role of a variety of adiponectin gene (ADIPOQ) at increasing the risk of colorectal cancer [50] or reduce its risk [40]. Results of a meta-analysis did not show any relationship between ADIPOQ change and colorectal cancer [51]. In another case-control study on 420 cancer patients with colorectal cancer and 555 control subjects, matched by age and sex factors, substantial association was found between Adipo R_1_ receptor and risk of colorectal cancer [36]. In a study on 62 cancer patients and 30 healthy individuals, in the age of 20–83 years, adiponectin levels were significantly lower (*P* < 0.001) in patients with oesophageal cancer [38]. This inverse relationship was significant in those blood samples that are collected over 5 years (*P*_trend_ = 0.031) [38]. While the results of two small case-control studies on pancreatic cancer, a conflicting correlation show between adiponectin levels and pancreatic cancer both in men and women [52, 53].

## 3. Discussion

The evidence from studies suggests a significant inverse relationship between serum adiponectin levels and risk of prostate, breast, endometrial, and colorectal cancer. Hormone levels are usually reduced in cancer patients [1, 30, 33, 43].

Adiponectin is inversely associated with obesity and insulin resistance and also it stimulates insulin sensitivity of peripheral tissues [1]. On the other hand, adiponectin reduction is associated with insulin resistance which leads to an increase in the levels and activity of IGF_1_. High concentrations of circulating IGF_1_ can increase the risk of breast cancer [2, 27]. Inverse relationship between adiponectin and breast cancer in post-menopausal women confirms the main effect of adiponectin in the pathogenesis of insulin resistance-related cancers such as breast cancer caused by obesity [27, 54]. IGF_1_ and insulin are effective in carcinogenesis through binding to their receptors which leads to an increase in cell proliferation and inhibit apoptosis in tissues which regulate the secretion of  vascular endothelial growth factor (VEFG) [15]. VEGF is secreted by breast cancer cells [55]. This factor is restrained by increasing the level of adiponectin through stimulating the ligand of peroxisome proliferator activated receptor *γ* (PPAR*γ*) [56]. Thus low levels of adiponectin leads to reduction in PPAR*γ* activity [57] and thus increase the risk of cancer. According to a study, the relationship between adiponectin and breast cancer risk is independent of the possible effects of IGF major system components, leptin, BMI, and menopausal status [18, 27].

Adiponectin plays an effective role in glucose metabolism, so that augmentation of glucose level lead to reduction of adiponectin. Importance of adiponectin on glucose and lipid metabolism is through the adiponectin receptors (R_1_ and R_2_) [58]. Relationship between obesity and breast cancer is mainly due to the estrogen receptors [2, 59]). Adiponectin suppresses the stimulation of growing cancer cells caused by estradiol [60]. Low level of adiponectin in obese patients and high level of estrogen increase the risk of breast cancer. While in another study the inverse relationship between hormone levels and breast cancer risk has been considered independent of estrogen levels during menopause [31]. In a case-control study on 70 women with cancer, low levels of adiponectin associated with obesity and lack of physical activity throughout life was considered as important risk factors for breast cancer as physical activity can decrease the cancer risk by 2–5 times. Physical activity can delay the first menstrual cycle by affecting a secretory production of ovarian hormones that reduce the risk of breast cancer [19].

Overweight and reduction of adiponectin concentrations are associated with endometrial cancer substantially by increasing estrogen and insulin resistance [33]. These hormones and obesity may each independently be associated with endometrial cancer [29]. Adiponectin increased adenosine monophosphate kinase (AMPK) and PPAR. AMPK and PPAR pathway activation leads to increasing insulin sensitivity and free fatty acid oxidation [61]. On the other hand, AMPK has direct effects on insulin resistance, cell proliferation, and apoptosis and it can interfere in tumor growth [29]. Thus reduction of AMPK can be effective in endometrial cancer. Adipo R_1_ receptors act mainly through AMPK pathways. Inhibitory effects of adiponectin on cancer cells are mainly affected by AMPK activity. Adipo R_1_ receptor plays an anticarcinogenic role [62].

 A research found that HMW has an inhibitory activity on prostate cancer cell growth and proliferation [45]. Furthermore, another study indicates a reduction of expression of adipo R_1_ and R_2_ receptors in prostate cancer tissues compared to benign prostatic hyperplasia (BPH) and healthy tissue [28]. Natural concentrations of adiponectin inhibit cancer cell growth, metastasis, and cancer cell lines and also inhibit dehydrotestosterone that stimulates cell proliferation [42, 62]. Adiponectin reduces the growth of androgen of prostate cancer cells through activation of AMPK up to 90% [63]. The hormone inhibits inflammation by inhibiting the activity of mature phagocytic macrophages which are associated with prostate cancer [64]. Obesity increases insulin production and insulin resistance. In addition, it can reduce adiponectin levels and AMPK activity which increase the risk of prostate cancer [28, 43]. 

IGF_1 _inhibits apoptosis and increases production of VEGF [41]. This factor is increased in patients with oesophageal cancer. Adiponectin has an inhibitory effect on transcription factor which is involved in regulation of VEGF [27]. This hormone also increased AMP kinase and also inhibits proliferation of colonic epithelial cells [65]. The reduction of the IGF binding protein (IGF_1_ BP) and increasing IGF_1_ in low levels of adiponectin concentrations can increase cell proliferation and inhibits apoptosis in colorectal cancer cells [47]. R_1_ and R_2_ adiponectin receptor expression in pancreatic *β*-cells expresses the relationship of adiponectin with the endocrine activity of pancreas [66]. This hormone can directly inhibit the growth and proliferation of pancreatic cancer cells [41]. On the other hand, positive correlation between adiponectin concentrations and risk of pancreatic cancer in two case-control studies with small sample volumes was found [52, 53]. Furthermore, the elevation of hormone secretion in cancer cells may be due to reducing hormone receptors [52]. However, in a study on 81 patients with pancreatic cancer and 81 healthy men and women R_1_ and R_2_ receptors on cancer cells were significantly expressed [52].

 Adiponectin receptor agonist that is called 355ADP was identified by isolating 149–166 peptide of adiponectin sequence. 355ADP adiponectin with similar behaviour to adiponectin was discussed as a recommended way to treat obesity, insulin resistance, and cancer [14].

 Adherence to Mediterranean dietary pattern and healthy dietary pattern can increase circulating adiponectin levels [67–69]. This association is shown not only in cohort and cross-sectional studies [68–70], but also in clinical trials [71]. Consumption of diet rich in fiber, low fat dairy products, whole grain, reducing energy intake from saturated fatty acids and refined grain, and increased consumption of fish and fish products and vegetable proteins may be effective to improve adiponectin levels [67, 68]. Low calorie diet with 10% weight reduction, in overweight and obese individuals, can influence on improvement of hormone levels [21, 72, 73]. As the importance of dietary pattern and dietary components on circulating adiponectin level, more investigations are recommended to clarify the effect of diet on adiponectin level. 

## 4. Conclusion

This study aimed to review the results of studies on the relationship between serum adiponectin levels and risk of cancer. Findings from different studies represent an inverse relationship between hormone levels and the risk of breast cancer [1, 27, 31], independent of BMI and menopausal status [20]. This association was observed in prospective and case-control studies [27, 31], both in pre- and post-menopausal women [20]. The risk of endometrial cancer in women who had high circulating adiponectin levels was less than those with lower adiponectin levels [34]. Also, it seems that the risk of prostate cancer and benign prostatic hyperplasia in men with low adiponectin level is higher than others [44, 46]. Several cohort and case-control studies suggest an inverse relationship between serum levels of adiponectin, genomic changes, and colorectal cancer risk [36, 48]. Result of a study on esophageal cancer indicates a low level of hormones in the patients [38]. It seems that there is an inverse relationship between plasma adiponectin and pancreatic cancer in men [41]. However, the results are conflicting in the men and women [52, 53].

## Figures and Tables

**Table 1:** Studies that investigated among association between levels of adiponectin and cancer.

Study	Type of study	Study comments	(OR, RR, HR)^1^	Adjusted variables	Results
Barb et al. [18]	Case-control	102 women with breast cancer/100 healthy women 50-51 years old	OR 3.62 (1.61–8.19)	Age, family history, menarche age, BMI, menopause age, marriage status	An inverse relationship between adiponectin levels and breast cancer pre- and post menopause

Jardé et al. [17]	Case-control	70 women with breast cancer/28 healthy women 25–65 years old	OR 0.2 (0.0–0.6)	Employment status, age at first pregnancy, smoking, alcohol consumption, OCP, hormone therapy, family history, breastfeeding, BMI	Inverse relationship between hormone concentration and breast cancer in pre- and postmenopause/abdominal obesity and physical inactivity increase the risk of cancer

Cust et al. [29]	Case-control	174 women with breast cancer/167 healthy women	OR 0.84 (0.71–0.99)	IGF Components, leptin, BMI, and socio- economic variables	Inverse relationship between adiponectin concentration and breast cancer in postmenopausal women

Otake et al. [30]	Prospective case-control	1477 women with breast cancer/2196 healthy women 30–55 years old	RR 1.3 (0.8– 2.1)^2^ 0.73 (0.55–0.98)^3^	Age, menopausal status, postmenopausal hormone use	No significant correlation between hormone levels and cancer risk

Tian et al. [1]	Hospital-based Case-control	244 women with breast cancer/244 healthy women	OR 0.55 (0.23–0.97)^3^	Age, BMI, waist to hip ratio	No significant correlation between hormone levels and risk of breast cancer in premenopause and a significant associated with breast cancer hormone in postmenopause

Kaklamani et al. [25]	Hospital-based Case-control	74 healthy women/74 women with breast cancer 30–82 years old	OR 0.23 (0.08–0.66)	Age, BMI, age at menarche, menopausal status, family history, insulin and leptin	0.77 Reduction in the cancer risk in women who were at the highest quartile of adiponectin levels

Körner et al. [26]	Prospective	248 women with endometrial cancer and 548 healthy women	RR 0.56 (0.36–0.86)	BMI	Stronger relationship between hormones and the risk of endometrial cancer in obese women

Tworoger et al. [31]	Case-control	87 women with endometrial cancer/132 healthy women 34–78 years old	OR 2.75 (1.16–6.54)^4^	Age, education, marriage status, smoking status, BMI, hormone replacement therapy	High energy intake and low adiponectin associated with increased risk of endometrial cancer

Treeck et al. [32]	Case control	117 women with endometrial cancer and 238 healthy women	OR 10.5 (4.18–26.35)	Age, BMI, diabetes, high blood pressure	11-fold increased risk of endometrial cancer in people who were at the lowest level of adiponectin compared with the highest level

Dal Maso et al. [33]	Case control	84 women with endometrial cancer/84 healthy women	OR 0.44 (0.24–0.81)	IGF, leptin, BMI, demographic-social variables	1 standard deviation increased adiponectin levels decreased 50% risk of breast cancer in women younger than 65 years old. No significant association was found in individuals older than 65 years

Soliman et al. [34]	Nested case-control	698 men with BPH/ 705 healthy men	OR 0.65 (0.47–0.87)	BMI, alcohol consumption, age	People with the highest hormone concentration were in 35% lower risk for BPH

Körner et al. [26]	Case control	75 men with prostatic cancer/75 men with BPH/150 healthy men	OR 0.9 (0.1–0.82)	Age, BMI, alcohol consumption, smoking	73% lower risk of prostate cancer in those in the highest quartile hormone levels compared with those who were in the lowest quartile

Petridou et al. [35]	Prospective	645 men with prostatic cancer and 644 healthy men	HR 0.35 (0.14–0.86)	BMI, peptide C, type and grade	Inverse relationship between sex hormones and fatal prostate cancer (comparing quintile 1 and 5)

He et al. [36]	Case-control	30 healthy people and 62 patients with esophageal cancer	—	—	Low adiponectin levels in cancer patients (*P* < 0.001)

Beebe-Dimmer et al. [37]	Nested case-control	381 people with colorectal cancer and 381 healthy people	—	—	No significant correlation between serum adiponectin levels and colorectal cancer

Yıldırım et al. [38]	Nested case-control	311 male cases with pancreatic cancer, 50–69 years old/510 control subjects	OR 0.65 (0.39–1.07)	Smoking status, blood pressure, C-peptide	The lower the cancer risk in people who were in the highest quintile of serum hormone

Goktas et al. [39]	Prospective	18225 men in total that 179 of whom were diagnosed with colorectal cancer, aged 40–75 years	RR 0.48 (0.25–0.9)	BMI	Significant correlation between hormone levels and cancer risk—no significant relationship was observed after adjustment for potential confounding

Kaklamani et al. [40]	Case-control	420 people with colorectal cancer and 555 healthy people	OR 0.53 (0.35–0.81)	Age, sex	Significant relationship between adiponectin R_1_ receptor and risk of colorectal cancer

Stolzenberg-Solomon et al. [41]	Case-control	81 people with pancreatic cancer and 81 healthy people	OR 2.81 (1.04–7.59)	Age, sex, BMI, smoking status, alcohol intake, history of diabetes, leptin	Positive and significant correlation between hormone concentrations and risk of pancreatic cancer

^1^RR: relative risk; OR: odds ratio; HR: hazard ratio.

^2^OR in premenopausal women.

^3^RR and OR in postmenopausal women.

^4^OR results of low level of adiponectin in combination with ≥2500 kcal energy intake.
